# A content and quality analysis of free, popular mHealth apps supporting ‘plant-based’ diets

**DOI:** 10.1371/journal.pdig.0000360

**Published:** 2023-10-25

**Authors:** Jennifer J. Lee, Mavra Ahmed, Rim Mouhaffel, Mary R. L’Abbé

**Affiliations:** 1 Department of Nutritional Sciences, Temerty Faculty of Medicine, University of Toronto, Toronto, Ontario, Canada; 2 Joannah & Brian Lawson Centre for Child Nutrition, University of Toronto, Toronto, Ontario, Canada; Iran University of Medical Sciences, IRAN (ISLAMIC REPUBLIC OF)

## Abstract

There has been an increased emphasis on plant-based foods and diets. Although mobile technology has the potential to be a convenient and innovative tool to help consumers adhere to dietary guidelines, little is known about the content and quality of free, popular mobile health (mHealth) plant-based diet apps. The objective of the study was to assess the content and quality of free, popular mHealth apps supporting plant-based diets for Canadians. Free mHealth apps with high user ratings, a high number of user ratings, available on both Apple App and GooglePlay stores, and primarily marketed to help users follow plant-based diet were included. Using pre-defined search terms, Apple App and GooglePlay App stores were searched on December 22, 2020; the top 100 returns for each search term were screened for eligibility. Included apps were downloaded and assessed for quality by three dietitians/nutrition research assistants using the Mobile App Rating Scale (MARS) and the App Quality Evaluation (AQEL) scale. Of the 998 apps screened, 16 apps (mean user ratings±SEM: 4.6±0.1) met the eligibility criteria, comprising 10 recipe managers and meal planners, 2 food scanners, 2 community builders, 1 restaurant identifier, and 1 sustainability assessor. All included apps targeted the general population and focused on changing behaviors using education (15 apps), skills training (9 apps), and/or goal setting (4 apps). Although MARS (scale: 1–5) revealed overall adequate app quality scores (3.8±0.1), domain-specific assessments revealed high functionality (4.0±0.1) and aesthetic (4.0±0.2), but low credibility scores (2.4±0.1). The AQEL (scale: 0–10) revealed overall low score in support of knowledge acquisition (4.5±0.4) and adequate scores in other nutrition-focused domains (6.1–7.6). Despite a variety of free plant-based apps available with different focuses to help Canadians follow plant-based diets, our findings suggest a need for increased credibility and additional resources to complement the low support of knowledge acquisition among currently available plant-based apps. This research received no specific grant from any funding agency.

## Author summary

With the growing interest in and known health benefits of plant-based diets, we examined the content and quality of 16 popular, freely available plant-based diet apps. We evaluated the apps’ quality using two different scoring systems. While most apps provided plant-based recipes and meal plans, some had other functions such as food scanning, community forums, and sustainability assessments. First, according to the Mobile App Rating Scale, the apps performed well on functionality and visual appeal but scored lower on trustworthiness. Second, based on the App Quality Evaluation scale, the apps were less effective in helping users learn about plant-based diets but performed adequately in other areas, such as assisting users to develop skills. This study provides valuable insights for Canadian developers and users of plant-based diet apps. Our findings showed that free plant-based apps could be improved by ensuring that the information they provide comes from reliable sources and that they can effectively support users in learning about plant-based diets.

## Introduction

Many recent dietary guidelines have emphasized increasing the consumption of plant-based foods and diets for health and environmental benefits [1,2]. However, plant-based diets vary widely in definition, thereby confusing consumers on how they can readily use guidelines to change their diet behaviors. Some refer to plant-based diets as a strict exclusion of all animal products (e.g., vegan diet), while others refer to high consumption of plant-based foods (e.g., vegetables, fruits, cereals, and beans) with occasional consumption of some animal-based products (e.g., dairy, seafood) [3]. Other studies further distinguish plant-based diets based on the quality of plant-based foods for cardiometabolic health benefits [4,5]. For instance, high consumption of ‘less healthy’ plant-based foods (e.g., fruit juices, sugar-sweetened beverages, refined grains, sweets/desserts) has shown adverse associations with risk of type 2 diabetes [4] and coronary heart disease [5], while protective associations were seen with high consumption of ‘healthier’ plant-based foods (e.g., whole grains, fruits, vegetables, nuts) [4,5]. Besides the heterogeneity of plant-based definition, consumers have reported additional barriers to the adoption of plant-based diets, including lack of knowledge, skills, social stigma, and financial constraints [6].

Digital technology may easily provide nutrition information and enable users to translate dietary guidelines into easy and meaningful actions for a sustainable behavioral change. Specifically, mobile health (mHealth) apps have been shown to have the potential to build new skills, promote healthy nutrition behaviors, address resource-related barriers, and reach a wide range of populations [7–16]. For instance, many nutrition-related health apps are available as recipe managers and meal planners to help build cooking skills [12,15], mHealth apps for patients with diabetes allow tracking of foods, nutrients, exercise, and weight [14], and Dietary Approaches to Stop Hypertension (DASH) mHealth apps provide information specific to the DASH dietary pattern to share diet information, similar to a handout [17]. However, little is known about the content and quality of plant-based diet mHealth apps. Therefore, the objective of the present study was to conduct a content analysis using nutrition experts to review and assess the quality of free, popular mHealth apps that are described to help users consume plant-based foods and/or adopt plant-based diets.

## Methods

### Study design

A cross-sectional content and quality analysis of free, popular plant-based diet mHealth apps was conducted.

### Eligibility criteria

Apps were included if they were: free (i.e., with no download fee); had a high user rating (≥3 out of 5 on Apple App and GooglePlay stores); had a high number of user ratings as a popularity indicator (≥100 ratings combined between Apple App and GooglePlay stores); available on both Canadian Apple App and GooglePlay stores; and described as an app to help identify or consume plant-based foods or follow a plant-based diet. Since the “plant-based” term remains vaguely defined, diets referring to the exclusion of animal-based products (i.e., vegetarian and vegan diets) were also used to define plant-based as part of the eligibility criteria in this study. Apps were excluded if they were paid-to-use; had low average user ratings (<3 out of 5 on either Apple App or GooglePlay store); had a low number of user ratings (<100 ratings on both Apple App and GooglePlay stores); only available on Apple App or GooglePlay store; non-English; non-food or nutrition-related Apps (e.g., gaming, dating); or did not include plant-based foods or diets as the primary focus (e.g., ethnic foods, weight loss).

### Information sources

Apple App and GooglePlay stores were searched on December 22, 2020.

### App search strategy

Apple App and GooglePlay stores were searched using pre-selected search terms: “plant-based,” “vegetarian”, “vegan”, “healthy eating”, and “healthy diet.” The top 100 returns for each search term were collected in the initial sample.

### App selection process

After the removal of duplicates, apps were evaluated against the inclusion and exclusion criteria over two stages. During the first stage, searched apps were evaluated for eligibility based on fee options, average user ratings, number of user ratings, app names and shortened descriptions; then, during the second stage, the remaining apps were evaluated based on their detailed descriptions available on Apple App and GooglePlay stores.

### Data collection process

The eligible apps were downloaded to iPhones (version 11 and 12, Apple Inc., Cupertino, CA). Descriptive and technical characteristics about the app (e.g., name, operator system availability, version, affiliations, developer’s name), app popularity (e.g., user ratings, number of reviews), and app descriptions were collected from both Apple App and GooglePlay stores. Three dietitians/nutrition research assistants (JJL, MA, and RM) independently evaluated the apps between February and March 2021.

### App quality assessment

App quality was assessed using two validated mHealth assessment tools: Mobile App Rating Scale (MARS) [18] and the App Quality Evaluation (AQEL) scale [19]. MARS is a 23-item questionnaire that assesses the quality of mHealth apps [18]. The MARS rates apps using 2 scores: overall quality and subjective quality. Overall quality scores related to general mHealth apps are assessed based on 4 domains (user engagement, app functionality, aesthetics, and quality of information) on a scale of 1 (inadequate) to 5 (excellent), then the scores from the 4 domains are averaged. App subjective quality scores related to personal recommendations and app value are assessed using 4 questions on a scale of 1 (the lowest rating) to 5 (the highest rating). A score of ≥4 was considered as high quality [18], 3–4 as adequate, and <3 as low.

AQEL is a 25-item questionnaire that evaluates nutrition mHealth app qualities based on 5 nutrition-focused domains [19]: potential to change behavior; support of nutrition knowledge acquisition; potential to develop nutrition-related skills; app technical functions; and purpose (i.e., consistency with the app description on app stores). Additional questionnaires are available to assess the appropriateness of the target age group (e.g., children, adults) and the type of target audience (e.g., individuals seeking help for a medical condition, people seeking weight loss support). Responses from each question are added by the AQEL assessment domain then converted into a scale of 0 to 10, where 0 represents the lowest quality and 10 represents the highest quality. Overall quality scores were obtained by calculating the mean score of the five AQEL main domains. A score of 8–10 was considered high [17], 6–8 as adequate, and <6 as low. Prior to rating the apps, all reviewers were trained on the MARS and AQEL scoring tools.

### Privacy and security setting assessment

**Table 1** shows the questionnaire used to assess privacy and security settings. Using a modified Online Trust Alliance survey for mHealth apps [20], a total of nine binary questions (i.e., yes or no) were used to assess privacy and security settings of the included apps. There were three questions on the availability of privacy policy, two questions on the accessibility of privacy policy, one question on data gathering, two questions on data sharing, and one question on data security.

**Table 1:** Privacy and security setting assessment questionnaire.

Category	Questions
**Availability**	Is there privacy information available?
	Is the privacy information available without the need to download the app? (e.g., app store, via a link to the privacy policy or the app’s website)
	Is the privacy information available within the app?
**Accessibility**	Is there a short form notice (in plain English) highlighting key data practices which are disclosed in detail in the full privacy policy?
	Is the privacy policy also available in French?
**Data gathering** ^*^	Does the app collect "Personally Identifiable Information"?
**Data sharing** ^*^	Does the app share users’ data with a 3^rd^ party?
	Is the app ad-supported? If yes, does it allow for advertising preference management in settings?
**Data security** ^*^	Does the app say how the users’ data security is ensured? (e.g., encryption, firewall system)

The questionnaire was developed using the Online Trust Alliance survey for mHealth apps [20]. Responses to all questions were binary (i.e., yes or no), unless privacy information was not available.

*Privacy information was used to respond to data gathering, data sharing, and data security questions.

### Statistical analysis

The included apps were categorized based on the primary app objective. App quality was assessed by three reviewers using the MARS and AQEL tools; scores were averaged and analyzed overall and by app type. The agreement of MARS and AQEL scores between the three assessors was examined using the intra-class correlation coefficient for two-way random, absolute inter-rater reliability (ICC_3,3_)_._ ICC ratio <0.50 indicated poor reliability, 0.50–0.75 as moderate reliability, 0.76–0.90 as good reliability, and >0.90 as excellent reliability [21]. All statistical analyses were conducted using RStudio (version 1.4.1106, RStudio, PBC). Statistical significance was considered at p<0.05.

## Results

### App selection

**Fig 1:**
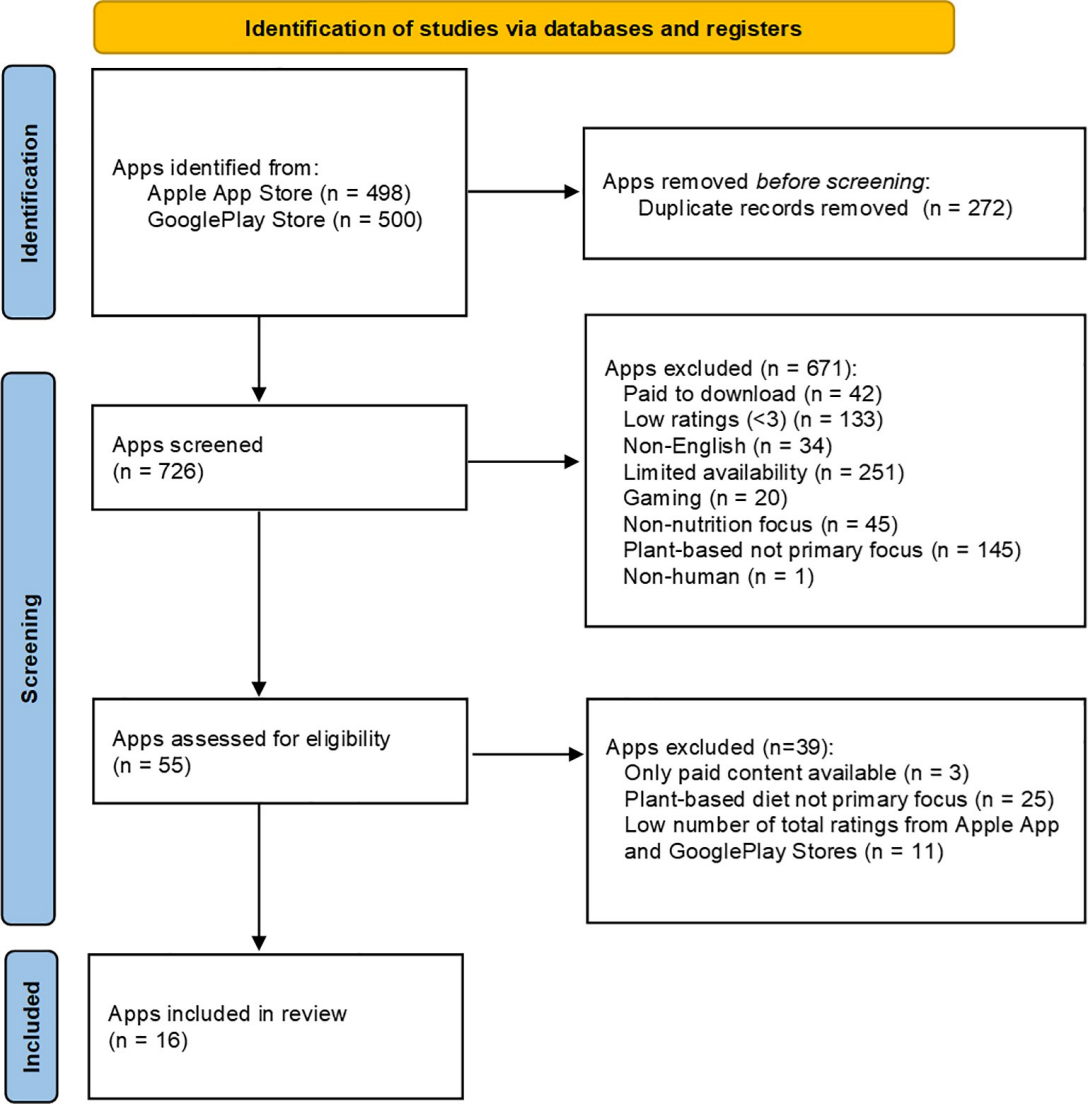
Flow diagram of free and popular plant-based app search and selection on Apple App and GooglePlay stores. Of the 726 apps screened (after the removal of duplicates), 671 were excluded based on fee options, average user ratings, app names, and shortened descriptions reported on Apple App and/or GooglePlay stores. The remaining 55 apps were reviewed using detailed descriptions available on Apple App and GooglePlay stores. A total of 16 plant-based apps met the inclusion criteria and qualified for further analysis.

**Fig 1** shows the flow diagram of the app selection. Of the 998 apps identified, 272 duplicates were removed. Apps were excluded based on fee options, average user ratings, number of user ratings, app names, and shortened descriptions (n = 671), then were further excluded using detailed descriptions on Apple App and/or GooglePlay (n = 39). Sixteen apps met the eligibility criteria for assessment.

### App characteristics

**Table 2** shows a summary of the characteristics of all included apps (**S1 Table** shows the summary of features, theoretical background and strategies, and other settings of all apps). The mean±SEM ratings of the included apps were 4.6±0.1 Based on the primary focus of the app descriptions, the included apps (n = 16) were categorized into one of five types: recipe managers and meal planners (n = 10), food scanners (n = 2), vegan community builders (n = 2), restaurant identifier (n = 1), and sustainability assessor (n = 1). All the included apps indicated to be appropriate for children older than 4 years of age (n = 12) and adolescents older than 12 years of age (n = 4); however, all 16 apps appeared to be only suitable for adults (≥18 y) based on the difficulty level of the provided information, required cognitive abilities, and/or required skills. Although all apps were free to download, 7 apps used two-tiered payment systems, offering additional features available at a fee (from $2.99 one-time fee to $67.99/y). Thirteen apps were updated within the last four months of the assessment (October 2021), while others had not been updated for as long as 2 years (since January 2019). Fourteen apps were developed by private groups or other non-government organizations, while 2 apps did not specify the developers’ affiliations. Eight apps were offered in languages other than English, 7 apps were offered in French, and 1 app offered as many as 138 additional languages.

**Table 2:** Characteristics of examined free popular plant-based apps.

App Name	App Version	Developer	Available Language(s)^a^	Additional Subscription Fee	Age Ratings^b^	App Type	Notable App Features
21-Day Vegan Kickstart	2.3.0	Physicians Committee for Responsible Medicine	EN + 1	None	>4 y	Recipe Manager or Meal Planner	• Provides a 21-day plant-based diet program with set meal plans, recipes (with nutrition info), and demo videos. • Has videos on health benefits of plant-based diet.
abillion	1.7.9	abillionveg, Inc.	EN + 1	None	>4 y	Community Builder	• Hosts a social platform for users interested in or practice a vegan lifestyle. • Provides information vegan products, restaurants, and other lifestyle information.
Food Book Recipes	29.0	Hitbytes Technologies	EN, FR + 10	$2.99 one-time	>12 y	Recipe Manager or Meal Planner	• Can save and organize recipes, showing level of difficulty, cooking time, and nutritional information. • Shows the proportion of vegan recipes in customized meal plans.
Kitchen Stories Recipes	15.3.2	AJNS New Media	EN, FR + 10	$29.99/y	>4 y	Recipe Manager or Meal Planner	• Provides different types of recipes with demo videos, level of difficulty, cooking time, and nutrition info; can filter for plant-based recipes.
Mary’s Recipes	3.6	Maria Kardakova	EN	$67.99/y	>4 y	Recipe Manager or Meal Planner	• Shows recipes and weekly meal plan options using recipes in the system—meal plans show intakes of food groups. (e.g., fruits & vegetables) and some nutrients (e.g., fiber). • Recipes can be automatically filtered based on dietary preferences (e.g., vegan only, meat free).
Mealime	4.8.3	Mealime Meal Plans Inc.	EN	$64.99/y	>4 y	Recipe Manager or Meal Planner	• Shows recipes (with cooking time) and weekly meal plan options using recipes in the system. • Recipes can be automatically filtered based on dietary preferences (e.g., vegetarian, pescatarian). • Shows food waste saved from meal plans.
Open Food Facts	3.4.3	Stephanie Gigandet	EN, FR +138	None	>4 y	Food Scanner	• Displays ‘vegetarian’ and ‘vegan’ status of packaged foods based on ingredients.
Quit Meat	1.05	AnnapurnApp Technologies UG haftungsbeschrankt	EN, FR + 2	None	>4 y	Sustainability assessor	• Estimates reduction in water and greenhouse gas emissions based on reported decrease intakes of animal-based foods.
Recipe Calendar	2.15	Vsevold Mayorov	EN + 4	$25.49/y	>4 y	Recipe Manager or Meal Planner	• Shows recipes and allows for making weekly meal plans—some pre-set meal plans show proportion of plant-based to animal-based foods. • Provides recipes (some as a link to websites) with total energy content, number of servings, cooking time, allergens, and cost (for some).
SideChef	5.3.0	SideChef Group Ltd.	EN	$66.99/y	>12 y	Recipe Manager or Meal Planner	• Provides different types of recipes with some demo videos, cooking time, and nutrition information; can filter for plant-based recipes. • Includes demo videos on cooking skills and tips. • Shows blog posts on nutrition and health.
Sirved	4.0.20	Sirved Mobile Solutions Inc.	EN, FR + 14	None	>4 y	Restaurant Identifier	• Displays menus of restaurants & highlights available vegetarian or vegan items.
Spoonful	2.9.0	Spoonful Inc.	EN	None	>4 y	Food Scanner	• Shows ‘vegetarian,’ ‘vegan,’ or ‘pescatarian’ compliant packaged foods based on ingredients
Tasty	2.50	BuzzFeed	EN	None	>4 y	Recipe Manager or Meal Planner	• Provides recipes with demo videos, cooking time, and nutrition info—allows for an automatic filter for only vegetarian recipes. • Provides cooking and nutrition tips.
Vegan Amino	1.8.35	Narvii Inc.	EN, FR + 7	None	>12 y	Community builder	• Hosts a social platform for users interested in or practice a vegan lifestyle.
Vegg’up	1.29.8	VEGG’UP	EN, FR	$36.99/y	>4 y	Recipe Manager or Meal Planner	• Shows vegetarian or vegan recipes with health information on vegan diets.
Yummly	6.4.2	Yummly	EN	None	>12 y	Recipe Manager or Meal Planner	• Provides different types of recipes with some demo videos, cooking time, and nutrition information. • Recipes can be automatically filtered based on dietary preferences (e.g., vegetarian, pescatarian). • Provides cooking and nutrition tips.

n = 16.

^a^Included apps were assessed for availability in English (EN), French (FR), and other languages (presented as a total number).

^b^The reported age ratings were obtained from the Apple App store.

Thirteen apps offered recipes on their platforms, with all 13 apps presented recipe images, 5 apps provided video demonstrations for select recipes, 8 apps showed nutrition information or nutrient analysis (e.g., total energy, macronutrient per serving), 8 apps showed total cooking time, 2 showed the level of difficulty of recipes, and 1 app showed recipe cost per serving (for select recipes only). However, the quality of food images and videos varied across apps. Food safety guidelines were poorly followed in videos of recipes and cooking demonstrations (e.g., demonstrators wearing nail polish and/or rings).

Six apps offered customizable meal planning options, where 1 app showed nutrition information planned per day and week (as the number of food group servings per day and week), and 1 app showed potential food waste savings from planned meals. Eight apps offered the ability to create grocery lists from selected recipes; however, no app showed any cost related to customized grocery lists nor the ability to set budgets. Fifteen apps offered nutrition education, where 10 apps focused on general cooking tips, 9 apps focused on nutrition education related to plant-based foods and/or diets, and 2 apps focused on the environmental benefits of plant-based foods and/or diets. Two apps offered social support for users through an online community to help adopt plant-based diet and other plant-based lifestyle behaviors (e.g., using vegan makeup products). Three apps showed restaurants with plant-based options to help users easily identify and plan meals outside of the home.

Numerous theoretical background/strategies (identified using the MARS app classification system [18]) were used to achieve the primary objectives of the apps. Fifteen apps used information/education, 9 apps used advice/tips/strategies/skills training, 6 apps used dietary assessment, 4 apps used monitoring/tracking, 6 apps used feedback (from developers or other users), 4 apps used goal setting, and 2 apps used cognitive behavioral therapy to achieve the app’s primary objectives.

Customizable features and settings were examined to assess the usability of the apps. Nine apps allowed for customization of various plant-based settings (e.g., vegetarian, vegan, pescatarian, flexitarian), dietary restrictions, allergies, and/or other food preferences. Three apps collected household composition or required serving size information, but only one app automatically adjusted the serving sizes of the recipes based on the household composition. Eleven apps offered information on non-plant-based foods and diet; however, only five included filter settings to remove the non-plant-based information. All apps allowed sharing options through e-mails, text messages, or other social medial platforms (e.g., Facebook, Twitter, WhatsApp). Four apps included app communities, where users could engage freely with others on the app. Fifteen apps required users to create a profile and a log-in username; however, no app offered an additional passcode system to access the app once logged on. Seven apps offered reminder options through phone or e-mail. Fifteen apps required Wi-Fi access to function. Two apps were linked with Siri to provide hands-free use option, and one app turned app usage into donations to promote usage and participation.

### App quality assessment

**Fig 2:**
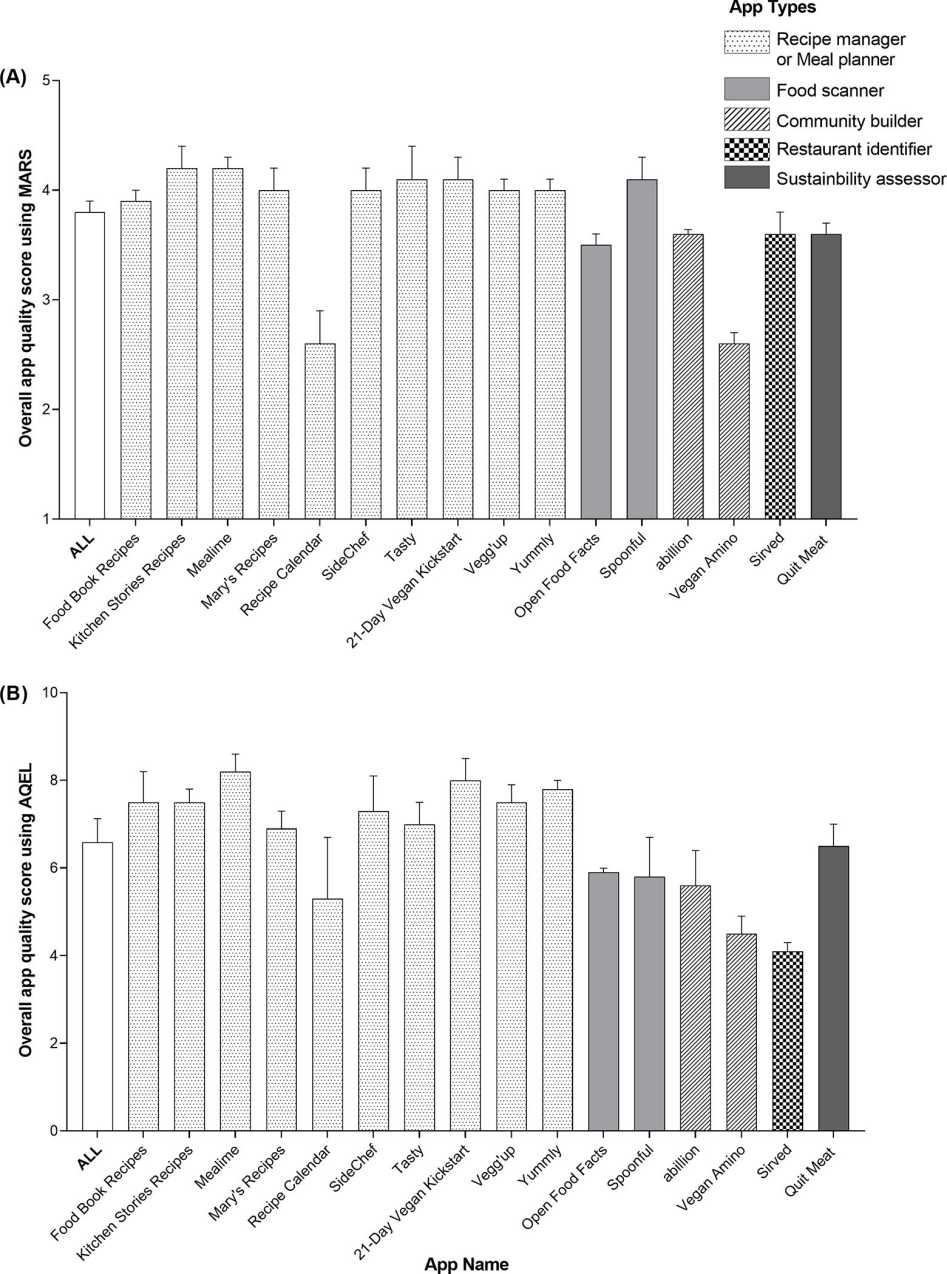
Overall app quality scores of plant-based mHealth apps using MARS and AQEL. Three dietitians/nutrition research assistants independently evaluated the quality of 16 plant-based apps between January and March 2021 using **(A)** MARS and **(B)** AQEL. Values represent means and SEM. Based on the primacy focus of the app descriptions, apps were categorized into one of 5 types (Recipe managers or Meal planners; Food scanners; Community builders; Restaurant identifier; Sustainability assessor. **(A)** Overall app quality score according to MARS represents the mean scores of the MARS four main categories (Engagement, Functionality, Aesthetics, and Information), and the scores ranged from 1–5. Intra-class coefficient (ICC_3,3_) of 0.909 [95% CI: 0.837, 0.952] indicated excellent agreement between the assessors. **(B)** Overall app quality score according to AQEL represents the mean scores of the AQEL five main categories (Behavioral change potential, Support of knowledge acquisition, Skill development, App function, and App purpose), and the scores ranged from 0–10. ICC_3,3_ of 0.884 [0.832, 0.922] indicated good agreement between assessors. Abbreviations: AQEL, App Quality Evaluation; MARS, Mobile App Ratings Scale; N/A, Not applicable.

**Fig 2** shows the overall MARS and AQEL scores overall and for each app, **Table 3** shows the summary of the MARS and AQEL scores overall and by app type, and **S2 Table** shows the scores for each individual app.

**Table 3:** Summary of the MARS and AQEL scores overall and by app type.

	Overall (n = 16)	Recipe Manager or Meal Planners (n = 10)	Food Scanners (n = 2)	Community Builders (n = 2)	Restaurant Identifier (n = 1)	Sustainability Assessor (n = 1)	ICC_3,3_ [95% CI]^a^
App Popularity^b^							
App ratings (range: 0–5)	4.6±0.1	4.7±0.1	3.9±0.3	4.6±0.1	4.7	4.5	
Total number of ratings	23,908±1,2763	37,191±19,544	1,357±559	3,179±580	277	1,270	
MARS (range: 1–5)							0.909 [0.837, 0.952]
Overall app quality	3.8±0.1	3.9±0.1	3.8±0.3	3.1±0.5	3.6	3.6	
Engagement	3.6±0.1	3.7±0.1	3.2±0.4	3.6±0.1	3.0	3.4	
Functionality	4.0±0.1	4.1±0.2	4.3±0.2	3.2±0.6	4.2	3.7	
Aesthetics	4.0±0.2	4.2±0.3	3.9±0.2	2.7±1.1	3.8	4.1	
Information	3.5±0.1	3.6±0.1	3.7±0.5	3.0±0.5	3.4	3.3	
App subjective quality	3.5±0.2	3.9±0.3	3.6±1.0	2.4±0.7	2.7	2.8	
AQEL (range: 0–10)							0.884 [0.832, 0.922]
Overall app quality	6.6±0.3	5.1±0.6	5.8±0.04	7.3±0.2	4.1	6.5	
Behavioral change potential	6.9±0.4	7.7±0.4	6.8±0.3	4.5±1.1	5.3	5.6	
Support of knowledge acquisition	4.5±0.4	4.3±0.4	4.7±0.5	5.6±0.01	2.1	6.7	
Skill development	6.1±0.7	8.2±0.4	3.7±0.02	3.3±1.5	0.4	4.1	
App function	7.4±0.4	8.0±0.4	6.8±0.4	5.8±2.0	5.3	7.7	
App purpose	7.6±0.3	8.0±0.3	7.2±0.5	6.1±1.1	7.2	8.3	
Suitability for the target audience							0.877 [0.827, 0.915]
Adults (18–64 y)	9.5±0.1	9.6±0.1	9.8±0.2	9.0±0.3	9.7	9.3	
Older adults (≥ 65 y)	9.0±0.1	9.2±0.1	9.0±0.3	8.2±0.5	9.0	8.7	
Individuals with food allergies or dietary restrictions	4.1±0.3	5.0±0.4	3.4±1.8	1.7±1.2	3.8	2.1	
Individuals shopping for food	5.8±0.4	5.8±0.3	8.4±0.4	3.4±0	N/A	N/A	
Individuals seeking recipes or meal ideas	5.8±0.8	6.1±0.3	N/A	4.4±0.2	N/A	5.8	
Individuals seeking guidance for restaurant eating	3.9±0.3	N/A	N/A	3.6±1.1	4.6	N/A	
Individuals seeking nutrition education	3.6±0.4	3.7±0.5	5.2±1.0	2.9±1.3	N/A	3.3	

Three dietitians/research assistants independently evaluated 16 plant-based apps between January and March 2021. Values represent means±SEM. ^a^Intra-rater reliability among the research assistants was assessed using two-way random, absolute intra-class coefficient (ICC_3,3_) and the 95% confidence interval (CI).

^b^App popularity was determined using the App ratings and the total number of ratings obtained from Apple App and GooglePlay stores on December 22, 2020. Abbreviations: AQEL, App Quality Evaluation; MARS, Mobile App Ratings Scale; N/A, Not applicable.

The MARS (scale: 1 to 5) revealed adequate overall app quality (mean±SEM: 3.8±0.1) and subjective quality score (3.5±0.2). Domain-specific assessment showed high functionality (4.0±0.1) and aesthetic scores (4.0±0.2), but lower engagement (3.6±0.1) and information (3.5±0.1) scores. In the information domain, the credibility score was low, with a mean score of 2.4±0.1, and only one app has been tested for its effectiveness using an intervention study with a sample of nurses [22]. Although MARS scores based on app type did not show any type to have high overall or subjective app quality scores, functionality and aesthetic scores were rated as high for recipe and meal planner apps (4.1±0.2 and 4.2±0.3, respectively). Assessment of individual app scores revealed 6 apps, all recipe and meal planners, to have high overall subjective quality scores. Excellent inter-rater reliability was observed for MARS assessment scores (ICC_3,3_: 0.909 [95% CI: 0.837, 0.952]).

The AQEL (scale: 0 to 10) revealed adequate scores in behavioral change potential (mean±SEM: 6.9±0.4), skill development (6.1±0.7), app function (7.4±0.4), and app purpose (7.6±0.3); but a low score in support of knowledge acquisition (4.5±0.4). Scores for suitability by target audience age groups were high (i.e., adults: 9.5±0.1, older adults: 9.0±0.1); however, scores for suitability for audience types were low (e.g., individuals with dietary restrictions: 4.1±0.3, individuals seeking nutrition education: 3.6±0.4). Assessment of scores by app types revealed that recipe and meal planner apps have high skill development scores (8.2±0.4), while food scanner apps revealed high scores in suitability for individuals shopping for food (8.4±0.4). Assessment of individual app scores showed 6 recipe and meal planner apps with high scores in behavioral potential scores and 7 recipe and meal planner apps with high skill development scores. Good inter-rater reliability was observed for AQEL quality assessment scores (ICC_3,3_: 0.884 [0.832, 0.922]) and suitability for target audience scores (ICC_3,3_: 0.877 [0.827, 0.915]).

### Privacy and security

**S3 Table** shows summary of the accessibility and availability of privacy and security information of the included apps. Fifteen apps offered a privacy policy statement in English accessible through the Apple App and/or GooglePlay stores (i.e., permits access without downloading the app). Three apps included a shortened version of the privacy policy statement in plain English. On the detailed versions of the privacy policy statements, fifteen apps indicated a collection of personally identifiable information, with nine apps stating sharing the users’ data with a 3^rd^ party while two apps had no mentions. Detailed versions of privacy statements of five apps indicated how users’ data security is ensured (i.e., encryption, authentication).

## Discussion

The present study assessed the content and quality of free, popular ‘plant-based’ mHealth apps for their ability to help users increase consumption of plant-based foods and/or adopt plant-based diets. We found 16 apps (10 recipe managers and meal planners, 2 food scanners, 2 vegan community builders, 1 restaurant identifier, and 1 sustainability assessor) that met the eligibility criteria based on app titles and descriptions. All included apps were indicated for use by a wide range of age groups; however, all apps appeared to be most suitable only for adults (≥18 y). App quality scores assessed using MARS and AQEL tools revealed adequate overall quality scores with high potential to change behaviors and support skill development for some recipe manager/meal planner apps. However, overall low scores were observed in knowledge acquisition potential, in part, due to the low credibility of the developers.

Our findings showed that skill development and behavioral change potential scores were the most commonly high scored domains with high scores shown in more than half of the recipe manager or meal planner apps. As plant-based protein foods (i.e., beans, legumes) are less frequently consumed in the Western diet [23,24], food handling and cooking skills may be helpful in overcoming some of the perceived barriers in adopting plant-based diets. The most significant perceived barriers reported in adopting plant-based diets included the enjoyment of meat and unwillingness to alter eating habits [25]. Considering plant-based protein foods typically require processing steps for human consumption, recipe-based apps can encourage users to try new foods and help users develop food skills to support a long-term, sustainable behavior change. Additional information on recipes (e.g., cooking time, necessary tools/equipment, difficulty levels, video demonstrations, nutritional information) and other customizable features (e.g., weekly reminders, dietary preferences) can further encourage users to make dietary changes. Interestingly, only one app that consistently received high quality ratings using both MARS and AQEL (i.e., 21-day Vegan Kickstart) incorporated cognitive behavioral therapy and interactive feedback from developers. Behavioral theories (e.g., Behavioral Intervention Technology Framework [26], Transtheoretical Model of Behavior [23]) have been shown to facilitate long-term behavioral changes; and customized feedback from developers may better engage users to support behavioral changes. Evidence-based mHealth apps grounded in behavioral theories with customizable feedback options may be helpful in supporting long-term, population-level behavioral changes using digital technology.

Although all included apps provided nutritional information and/or educational content to achieve their primary objectives, they showed poor ability to support knowledge acquisition. Nutrition mHealth apps have the potential to address the health and information barriers in consuming ‘healthy’ plant-based foods and diets by providing clear descriptions of plant-based diets [27] and helping determine ‘high quality’ plant-based foods [28]. Yet, the examined apps showed overwhelming, irrelevant, and/or inaccurate content often with poor readability and lack of credible sources cited, which contributed to low knowledge acquisition scores. In particular, the credibility scores were low due to the poor nutrition credibility of app developers (i.e., private and small non-governmental organizations) and the lack of evidence on the effectiveness of apps. Thus, there is a need for credible and reputable organizations (e.g., government agencies, researchers, health organizations) to expand their scope of communication methods into the evolving field of digital health to fill this gap by providing clear, accurate, and reliable nutrition information. Further, as governments and health agencies develop and update dietary guidelines, consumer-friendly apps to support new guidelines or recommendations should be considered.

Although the apps focused on creating inclusive community environments, which may encourage the adoption and maintenance of plant-based diets, their benefits and risks should be carefully weighed. Some social barriers with reports of fear of ‘not fitting in’ have been identified as frequent barriers in adopting plant-based diets [25,29]. MHealth apps offering online social platforms can provide a unique opportunity to easily expand the social network of users by connecting them with others around the world [30]. However, similar to digital threats to most social networking websites [30], there are credibility, privacy, and security risks associated with social networking apps. With no legal responsibility for the credibility of information shared by other users on websites and apps [31], there was no clear verification process of nutrition information shared by other users on social networking apps, leaving users to verify all gathered information. Further, it is unclear if and how the app content is used by the end users. Although the apps reported suitability for a wide range of age groups, including children and adolescents, the lack of clarity on the interpretation of the app content can lead to adverse health behaviors such as early dieting and extreme weight loss behavior. Extra caution should be considered to assess app suitability for populations at risk to ensure that apps classified to be appropriate for children and adolescents are indeed so.

App functionality and aesthetic scores assessed using both MARS and AQEL were high for all sixteen apps, highlighting the significance of the technical characteristics in reaching a wide audience with mHealth apps. According to the Behavioral Intervention Technology framework, one of the main elements of designing effective eHealth and mHealth tools is considering the technical characteristics (i.e., how to better meet the needs of users and achieve the app’s goals) by addressing four factors: medium, complexity, aesthetics, and personalization [26]. In particular, aesthetics may help attract users’ attention as they explore different options before app use, and may increase acceptance and perceived credibility while they use the app. Researchers should ensure aesthetics are considered when developing apps to maximize acceptance and reach among the general public; however, information need to be clearly communicated to app users to help them easily determine the credibility of the apps.

Although most of the apps provided basic privacy information, the overall accessibility and quality of the information was poor. A previous study showed that the reading level of detailed privacy statements tends to be high at approximately 2 years of college training level [32], challenging the public to fully comprehend the detailed statements. Despite most apps collecting various personal information through personal profile creation and app usage, only about half of the apps provided information on data encryption to inform users about the protection of personal information. Consistent with similar studies on the availability of privacy statements on health websites [32], the poor availability and accessibility of privacy statements prior to the app download can dissuade users from seeking and reading the privacy information. Other mHealth apps have been shown to collect high-risk classifying information (e.g., full name, date of birth, health information) and sharing of the data with third parties while using ambiguous statements on their data sharing information [33]. Although the Personal Information Protection and Electronic Documents Act (PIPEDA) in Canada mandates the protection of personal information [34], the rapid developments in apps from organizations worldwide may limit compliance with national regulations. The adoption of national or international regulations specific for mHealth apps (e.g., HAS Good Practice Guidelines on Health Apps and Smart Devices [35]) by app providers (e.g., Apple App and GooglePlay stores) can be helpful in ensuring users are well protected regardless of the apps’ origin.

We used a systematic approach to analyze the content and quality of free and popular plant-based mHealth apps, there are some limitations to the present study. First, our inclusion criteria included apps with a primary focus of adopting plant-based foods, resulting in excluding apps that may have plant-based diets as a secondary focus. For instance, we excluded apps with a primary focus on weight loss or gain, management of medical conditions (e.g., diabetes), mobile counselling (e.g., dietetic services), and other more prescriptive plant-based food focused dietary patterns (e.g., Mediterranean, DASH). The excluded apps may also be helpful for users interested in adopting plant-based diets with more structured and rigid guidelines. Second, we excluded paid apps and other paid aspects of the included apps. Payment structure can help secure funds for consistent app updates to ensure information is up-to-date, address any technical issues, and address feedback from users. However, the lack of credible and functional free mHealth apps can be problematic in creating an equitable digital health environment. Funding support for research and development of publicly available apps can be helpful in creating equitable digital health environment. Third, the current study was assessed cross-sectionally, limited to examining popular apps identified on a one-day search based on user ratings and the number of ratings across Apple App and GooglePlay stores in Canada. Although our analysis focused on examining what users may be first exposed to at the time of the app search, numerous mHealth apps are available for downloads on Apple App and GooglePlay stores with constantly changing popularity ratings. The quality of the apps may change with updates as developers make modifications based on user feedback [7,8]. As of February 2023, 12 of the included apps had been updated several times since the initial evaluation and 2 have been removed. For healthcare providers interested in incorporating mHealth apps into their practice, familiarity with the apps will be helpful in providing the most up-to-date and individualized app recommendations for their clients. Although all apps evaluated in this study were rated highly by users (≥3 out of 5 ratings), the results of this study indicate the importance of assessing nutrition content and quality of the apps by nutrition professionals. Our findings highlight a research gap in nutrition mHealth apps in this area; however, additional quantitative and qualitative research from various stakeholders, including healthcare providers practicing in different areas and users of the apps are needed to better understand the functionality and usability of these apps.

## Conclusion

Many free plant-based mHealth apps with different focuses are currently available to help Canadians follow plant-based diets. The majority of the plant-based mHealth apps were recipe managers and/or meal planner apps, providing information and supporting skill development to help users consume more plant-based foods or adhere to plant-based diets. Despite having potential to drive behaviour change and support skill development among users, our quality assessment findings suggest there is a need for credible apps and additional resources to complement the low support of knowledge acquisition in plant-based mHealth apps.

## Supporting information

S1 Text Abbreviations of included free, popular plant-based apps. (PDF)

S1 Table Summary of features, theoretical background, and other settings of free, popular plant-based apps. (PDF)

S2 Table Summary of app popularity and quality scores of free, popular plant-based apps. (PDF)

S3 Table Summary of the accessibility and availability of app privacy and security information. (PDF)

S1 Data Dataset. (XLSX)
