# Supplementary material for: A content and quality analysis of free, popular mHealth apps supporting ‘plant-based’ diets
Source: PLOS Digit Health. 2023 Oct 25;2(10):e0000360. doi: 10.1371/journal.pdig.0000360 (PMC10599568; doi:10.1371/journal.pdig.0000360)
Supplement: S1 Text — (PDF) [file pdig.0000360.s001.pdf]

S1 Text. Abbreviations of included free, popular plant-based apps

AB: abillion
FB: Food Book Recipes
KS: Kitchen Stories Recipes
ML: Mealime
MR: Mary's Recipes: Meal Planner
OF: Open Food Facts – Product Scan
QM: Quit Meat - Eat less Meat
RC: Recipe Calendar - Meal Planner
SC: SideChef: Recipes + Meal Planner
SP: Spoonful: Food Scanner
SV: Sirved - Restaurant Menus
TA: Tasty
VA: Vegan Amino for: Vegannism
VK: 21-Day Vegan Kickstart
VU: Vegg'up, Vegetarian Recipes
YU: Yummly
