# Supplementary material for: A content and quality analysis of free, popular mHealth apps supporting ‘plant-based’ diets
Source: PLOS Digit Health. 2023 Oct 25;2(10):e0000360. doi: 10.1371/journal.pdig.0000360 (PMC10599568; doi:10.1371/journal.pdig.0000360)
Supplement: S1 Table — (PDF) [file pdig.0000360.s002.pdf]

S1 Table. Summary of features, theoretical background, and other settings of free, popular plant-based apps.

App Abbreviations	FB	KS	ML	MR	RC	SC	TA	VK	VU	YU	OF	SP	AB	VA	SV	QM
App Type	RM	RM	RM	RM	RM	RM	RM	RM	RM	RM	FS	FS	CB	CB	RI	SA
**App Features**																
Recipes	✓	✓	✓	✓	✓	✓	✓	✓	✓	✓			✓	✓		✓
Images	✓	✓	✓	✓	✓	✓	✓	✓	✓	✓			✓	✓		✓
Nutrition information	✓	✓		✓	✓*		✓	✓	✓	✓						
Total cooking time	✓	✓	✓		✓	✓	✓		✓	✓						
Level of difficulty	✓	✓														
Cost					✓*											
Save	✓	✓	✓	✓	✓		✓	✓	✓	✓			✓			
Ratings/feedback	✓	✓	✓		✓		✓		✓	✓			✓	✓		
Video demonstrations		✓*				✓*	✓*	✓*		✓*						
Add own recipe				✓	✓								✓	✓		
Meal Planners	✓		✓	✓	✓	✓		✓								
Nutrition information (per day or week)				✓												
Food waste savings			✓													
Grocery lists	✓	✓	✓	✓	✓		✓	✓	✓							
Cost																
Food Scanners											✓	✓				
Nutrition Education	✓	✓	✓	✓	✓	✓	✓	✓	✓	✓	✓	✓	✓	✓		✓
Cooking tips	✓	✓	✓	✓	✓	✓	✓	✓		✓				✓		
Related to plant-based food/diets	✓		✓			✓	✓	✓	✓				✓	✓		✓
Sustainability			✓													✓
Community-based (i.e., social support)													✓	✓		
Restaurant recommendations													✓	✓	✓	
**Theoretical Background and Strategies**[a]																
Dietary assessment		✓		✓						✓	✓	✓				✓
Feedback (from developers)			✓					✓								
Feedback (from other users)				✓									✓	✓	✓	
Information/Education	✓	✓	✓	✓	✓	✓	✓	✓	✓	✓	✓	✓	✓	✓		✓
Monitoring/Tracking			✓	✓				✓								✓
Advice/Tips/Strategies/Skills training	✓	✓		✓				✓	✓	✓			✓	✓		✓
Goal setting			✓	✓				✓								✓

Cognitive behavioral therapy								✓								✓
**Customizable Settings**																
Various plant-based settings (e.g., vegetarian, vegan, pescatarian)			✓	✓	✓	✓			✓	✓	✓	✓			✓	
Dietary restrictions and/or preferences			✓	✓	✓	✓			✓	✓	✓	✓			✓	
Household composition			✓						✓	✓						
Non-plant-based options	✓	✓	✓	✓	✓	✓	✓			✓	✓	✓			✓	
Ability to ‘hide’ non-plant-based options		✓	✓			✓	✓			✓						
Allows sharing (Facebook, Twitter, etc.)	✓	✓	✓	✓	✓	✓	✓	✓	✓	✓	✓	✓	✓	✓	✓	✓
Has an app community		✓										✓		✓	✓	
Requires login	✓	✓	✓	✓	✓	✓	✓	✓	✓	✓		✓	✓	✓	✓	✓
Sends reminders			✓	✓		✓		✓		✓				✓	✓	
Needs wi-fi access to function	✓	✓	✓	✓	✓	✓	✓	✓	✓	✓	✓	✓	✓	✓	✓	
Hands-free option (i.e., linked with Siri)	✓	✓														
App use into donations													✓			

Theoretical Background and Strategies were derived from the Mobile Application Rating Scale (MARS) App Classification section [1]. Abbreviations: CM, Community builders; FS, Food scanners; RI, Restaurant identifier; RM, Recipe managers or meal planners; SA, sustainability assesspr.
✓ indicates characteristics that are always present in the app.
✓* indicates characteristics that are often present in the app.

References

1. Stoyanov SR, Hides L, Kavanagh DJ, Zelenko O, Tjondronegoro D, Mani M. Mobile app rating scale: a new tool for assessing the quality of health mobile apps. JMIR mHealth and uHealth. 2015;3(1):e27. doi: 10.2196/mhealth.3422
