# Supplementary material for: A content and quality analysis of free, popular mHealth apps supporting ‘plant-based’ diets
Source: PLOS Digit Health. 2023 Oct 25;2(10):e0000360. doi: 10.1371/journal.pdig.0000360 (PMC10599568; doi:10.1371/journal.pdig.0000360)
Supplement: S2 Table — (PDF) [file pdig.0000360.s003.pdf]

S2 Table. Summary of app popularity and quality scores of free, popular plant-based apps

**App Abbreviations**	**FB**	**KS**	**ML**	**MR**	**RC**	**SC**	**TA**	**VK**	**VU**	**YU**	**OF**	**SP**	**AB**	**VA**	**SV**	**QM**
**App Type**	RM	RM	RM	RM	RM	RM	RM	RM	RM	RM	FS	FS	CB	CB	RI	SA
**App Popularity[a]**																
App ratings	4.4	4.75	4.65	4.75	4.65	4.5	4.8	4.5	3.75	4.6	3.8	4.45	4.8	4.55	4.7	4.45
Total number of ratings	1,743	33,420	23,490	657	7,389	4,929	179,431	289	878	120,301	2,607	107	1,881	4,476	277	1,270
**MARS[b]**																
Overall App quality	3.9±0.1	4.2±0.2	4.2±0.1	4.0±0.2	2.6±0.3	4.0±0.2	4.1±0.3	4.1±0.2	4.0±0.1	4.0±0.1	3.5±0.1	4.1±0.2	3.6±0.04	2.6±0.1	3.6±0.2	3.6±0.1
Engagement	3.4±0.1	3.8±0.4	4.0±0.1	3.9±0.1	2.9±0.3	3.7±0.2	4.0±0.2	3.4±0.1	3.9±0.2	4.0±0.1	2.9±0.2	3.6±0.1	3.5±0.1	3.7±0.3	3.0±0.3	3.4±0.2
Functionality	4.2±0.3	4.2±0.1	4.5±0.1	4.2±0.2	4.1±0.2	4.1±0.2	4.3±0.2	4.3±0.3	4.2±0	4.2±0.1	4.1±0.2	4.5±0.1	3.8±0.2	2.6±0.2	4.2±0.2	3.7±0.2
Aesthetics	4.2±0.2	4.8±0.2	4.6±0.2	4.3±0.4	2.3±0.3	4.6±0.2	4.2±0.5	4.3±0.3	4.3±0.3	4.4±0.3	3.7±0.2	4.1±0.5	3.8±0.2	1.7±0.3	3.8±0.2	4.1±0.1
Information	3.7±0.2	3.9±0.1	3.6±0.1	3.7±0.2	2.6±0.4	3.7±0.1	3.9±0.3	3.9±0.2	3.7±0.1	3.5±0.1	3.3±0.2	4.1±0.2	3.4±0.1	2.5±0.2	3.4±0.4	3.3±0.5
App subjective quality	3.3±0.7	3.9±0.7	4.6±0	4.4±0.3	1.8±0.6	4.1±0.3	3.8±0.9	4.0±0.7	4.5±0.3	4.6±0	3.3±0.3	3.9±0.9	3.0±0.3	1.8±0.3	2.7±1.0	2.8±0.3
**AQEL[c]**																
Overall App quality	7.5±0.7	7.5±0.3	8.2±0.4	6.9±0.4	5.3±1.4	7.3±0.8	7.0±0.5	8.0±0.5	7.5±0.4	7.8±0.2	5.9±0.1	5.8±0.9	5.6±0.8	4.5±0.4	4.1±0.2	6.5±0.5
Behavioral change potential	8.0±1.0	7.3±0.7	8.5±0.8	8.3±0.4	4.8±1.8	7.2±0.9	7.3±1.0	8.0±0.6	8.4±0.5	9.3±0.5	6.5±0.7	7.1±1.5	5.6±1.4	3.3±0.1	5.3±0.4	5.6±0.8
Support of knowledge acquisition	3.9±0.3	3.9±0.2	7.4±0.3	3.8±0.4	2.9±0.7	4.8±0.7	4.1±0.4	5.0±0.3	3.9±0.2	4.2±0.4	4.2±0.4	5.2±0.3	5.6±0.4	5.6±0.7	2.1±0.6	6.7±0.6
Skill development	8.5±0.7	8.9±0.6	8.9±0.6	7.4±1.3	5.6±2.6	8.2±1.3	8.9±0.6	8.9±0.6	7.8±1.3	10.0±0	3.7±0.7	3.7±2.3	1.9±1.9	4.8±0.8	0.4±0.4	4.1±0.8
App function	8.5±0.8	8.9±0.3	8.8±0.4	7.9±0.2	5.3±1.6	7.6±1.3	7.5±1.0	9.0±0.6	8.5±0.6	8.9±0.7	7.2±0.7	6.4±0.9	7.8±0.4	3.8±0.6	5.3±0.4	7.7±0.6
App purpose	8.9±0.3	8.3±0.6	7.2±0.3	7.2±0.3	8.0±0.3	8.9±0.3	7.2±0.6	8.9±0.3	9.1±0.7	6.7±0.3	7.8±0.9	6.7±0.7	7.2±0.7	5.0±0.9	7.2±0.6	8.3±0.7
Suitability for the target audience[‡]																
Adults (18 – 64 y)	9.7±0.3	9.7±0.3	9.3±0.3	9.7±0.3	9.3±0.3	9.7±0.3	9.7±0.3	10.0±0	9.3±0.7	9.7±0.3	9.7±0.3	10.0±0	9.3±0.7	8.7±1.3	9.7±0.3	9.3±0.7
Older adults (≥65 y)	9.3±0.3	9.0±0.3	9.3±0.3	9.3±0.3	8.7±0.3	8.7±0.3	9.0±0.3	9.7±0	9.3±0.7	9.3±0.3	8.7±0.3	9.3±0	8.7±0.7	7.7±1.3	9.0±0.3	8.7±0.7
Individuals with food allergies or dietary restrictions	2.1±1.5	5.4±1.1	6.7±0.8	5.8±0.8	4.6±0.4	5.0±0	4.6±0.4	5.0±1.9	5.8±1.7	4.6±0.4	2.1±0.8	4.8±1.7	2.9±1.5	0.4±0.4	3.8±0.7	2.1±1.5
Individuals shopping for food	5.0±0	5.7±0.5	6.3±0.7	6.3±0.7	5.8±0.8	5.8±0.8	5.8±0.8	4.6±0.8	5.0±1.4	7.5±0	8.0±0.8	8.8±1.2	3.4±1.1	3.4±1.1	N/A	N/A
Individuals seeking recipes or meal ideas	6.7±1.7	6.3±0.7	6.3±0.7	6.3±0.7	5.4±1.1	6.7±0.4	5.8±0.8	3.8±1.9	6.3±0.7	7.5±0	N/A	N/A	4.6±1.8	4.2±0.8	N/A	5.8±0.8
Individuals seeking guidance for restaurant eating	N/A	N/A	N/A	N/A	N/A	N/A	N/A	N/A	N/A	N/A	N/A	N/A	4.6±0.8	2.5±0	4.6±0.8	N/A
Individuals seeking nutrition education	2.1±0.8	2.5±1.2	5.0±0	2.1±1.5	1.7±1.1	4.2±0.8	4.2±0.8	5.8±1.7	4.2±0.8	5.4±0.4	6.3±0.7	4.2±2.2	4.2±1.8	1.7±0.8	N/A	3.3±1.7

Three dietitians/nutrition research assistants independently evaluated 16 plant-based diet apps between January and February 2021. Values represent means (standard error of mean). Good to excellent intra-rater reliability was observed among the research assistants ($ICC_{3,3}$ range: 0.87-9.09). [a]App ratings and total number of ratings presented in Apple iTunes and GooglePlay stores on December 30, 2020 were collected to assess App popularity. App ratings were collected from users using a 5-star system, 1 indicating low rating and 5 indicating high rating. [b]Mobile App Rating Scale (MARS) scores ranged from 1 (lower quality) to 5 (higher quality) with ≥4 indicating high quality [1]. [c]App Quality Evaluation (AQEL) scores ranged from 0 (lower quality) to 10 (higher quality) with ≥8 indicating high quality [2]. Abbreviations: CM, Community builders; FS, Food scanners; RI, Restaurant identifier; RM, Recipe managers or meal planners; SA, sustainability assessor.

References

1. Stoyanov SR, Hides L, Kavanagh DJ, Zelenko O, Tjondronegoro D, Mani M. Mobile app rating scale: a new tool for assessing the quality of health mobile apps. JMIR mHealth and uHealth. 2015;3(1):e27. doi: 10.2196/mhealth.3422

2. DiFilippo KN, Huang W, Chapman-Novakofski KM. A new tool for nutrition app quality evaluation (AQEL): development, validation, and reliability testing. JMIR mHealth and uHealth. 2017;5(10):e163. doi: 10.2196/mhealth.7441
