# Supplementary material for: A content and quality analysis of free, popular mHealth apps supporting ‘plant-based’ diets
Source: PLOS Digit Health. 2023 Oct 25;2(10):e0000360. doi: 10.1371/journal.pdig.0000360 (PMC10599568; doi:10.1371/journal.pdig.0000360)
Supplement: S3 Table — (PDF) [file pdig.0000360.s004.pdf]

A content and quality analysis of free, popular mHealth apps supporting ‘plant-based’ diets

Lee, J. J., Ahmed, M., Mouhaffel, R., L’Abbe, M. R.

S3 Table. Summary of the accessibility and availability of app privacy and security information.

**App Abbreviations**	**FB**	**KS**	**ML**	**MR**	**RC**	**SC**	**TA**	**VK**	**VU**	**YU**	**OF**	**SP**	**AB**	**VA**	**SV**	**QM**
**App Type**	RM	RM	RM	RM	RM	RM	RM	RM	RM	RM	FS	FS	CB	CB	RI	SA
**Availability**																
General availability	✓	✓	✓	✓	✓	✓	✓	✓	✓	✓		✓	✓	✓	✓	✓
Detailed statement on Apple App or GooglePlay	✓	✓	✓	✓	✓	✓	✓	✓	✓	✓		✓	✓	✓	✓	✓
Detailed statement within the App	✓	✓	✓	✓	✓	✓	✓		✓	✓		✓	✓	✓	✓	
**Accessibility**																
Shortened statement in plain English								✓	✓	✓						
Detailed statement in French									✓					✓		
**Data gathering**																
Collection of personal information	✓	✓	✓	✓	✓	✓	✓	✓	✓	✓			✓	✓	✓	✓
**Data sharing**																
Sharing of data with a 3rd party	✓	✓	✓			✓	✓	✓		✓				✓	✓	
Ad-supported	✓			✓	✓					✓						
Option to manage advertising preference	✓*									✓						
**Data security**																
Security measures for protecting personal data	✓					✓		✓		✓						✓

✓ indicates privacy and security information present for the app.

✓* indicates option available under premium settings (i.e., paid).

Abbreviations: CM, Community builders; FS, Food scanners; RI, Restaurant identifier; RM, Recipe managers or meal planners; SA, sustainability assessor.
